# Bionic Design and 3D Printing of Continuous Carbon Fiber-Reinforced Polylactic Acid Composite with Barbicel Structure of Eagle-Owl Feather

**DOI:** 10.3390/ma14133618

**Published:** 2021-06-29

**Authors:** Yunhong Liang, Chang Liu, Qian Zhao, Zhaohua Lin, Zhiwu Han, Luquan Ren

**Affiliations:** 1 The Key Laboratory of Bionic Engineering, Ministry of Education, Jilin University, Changchun 130025, China; liangyunhong@jlu.edu.cn (Y.L.); Liu_Chang19@mails.jlu.edu.cn (C.L.); zwhan@jlu.edu.cn (Z.H.); lqren@jlu.edu.cn (L.R.); 2 School of Mechanical and Aerospace Engineering, Jilin University, Changchun 130022, China; linzhaohua@jlu.edu.cn

**Keywords:** continuous carbon fiber, 3D printing, bionic feather structure, mechanical property

## Abstract

Inspired by eagle-owl feather with characteristics of light weight and high strength, the bionic continuous carbon fiber-reinforced polylactic acid composite with barbicel structure was successfully 3D printed. Under the action of external load, angles between barbicels and rachis structure of eagle-owl feather decreased, which consumed a part of energy and built structure base of bionic feather structure model with a certain arrangement angle of continuous carbon fiber. Variation of bionic structure model design parameters significantly affected the mechanical properties of the 3D printing bionic composites. The relatively low continuous carbon fiber content on tensile force direction restricted enhancement of tensile strength of bionic composite. However, attributed to different angle arrangement of continuous carbon fiber, the propagation of cracks in bionic composite was hindered, exhibiting high impact resistance. The effective and feasible bionic feather design and 3D printing of continuous carbon fiber-reinforced polylactic acid composite extended the corresponding application in the areas with high impact loads.

## 1. Introduction

Attributed to the excellent tensile strength [1,2,3,4], impact toughness [5,6], fatigue resistance [7,8] and high specific stiffness [9], fiber-reinforcement resin matrix composites were widely used as heat-resisting material [10], lightweight material [11], wear-resistant materials [12,13] and bio-based material [14,15,16].

Compared with glass fiber [17], basalt fiber [18], agamid fiber [19] and natural fiber [20,21,22], carbon fiber [23] has the advantages of higher specific strength, specific modulus, smaller density, stronger fatigue resistance and high temperature resistance. Based on the advantage of carbon fiber, it was used as a reinforcing material and composites with epoxy [24], ceramic [25] and metal [26]. Polytechnic acid (PLA) [27,28] was a biodegradable material and widely used in additive manufacturing, which enhanced environmental friendliness. Carbon fiber-reinforced PLA resin had the characteristics of light-weight and high strength and environmental friendliness, which were widely used in aerospace [29,30,31,32], transportation [33,34] and human prosthetics [35,36]. Up to now, short carbon fiber was treated as reinforcement in PLA resin matrix, which mixed and heated short carbon fibers and PLA particles. However, the directional arrangement of short carbon fiber restricted the reinforcing effect. Fused Deposition Modeling (FDM) 3D printing can realize high precision molding of PLA. Attributed to the length and the quantity of short carbon fiber, short carbon fiber-reinforced PLA composite is hard to achieve directional arrangement via FDM 3D printing. Compared with the short carbon fiber, continuous carbon fiber is applicative in FDM. The PLA wire rod and the continuous carbon fiber can be squeezed into the heating nozzle together, leading to the existence of PLA on the carbon fiber surface. Even though FDM realized the continuous carbon fiber-reinforced PLA composite with high precision molding and extended the application field, the mechanical strength enhancement can be investigated further.

Facing the efficiency and cost demands of application of carbon fiber-reinforced resin composite, bionics structure provided a new method to enhance mechanical strength of composites. Bird feathers generated thrust and lift when encountering aerodynamic forces, which realized fly [37]. The light weight and high strength of feathers can be treated as a bionic model. The rachis structure and feather barbicels structure of feathers are not arranged vertically. Compared with samples arranged at 0°/90°, the samples arranged at ±45° had stronger fatigue endurance by changing the arrangement of fibers [38]. The composites with a special angle braid owned high ultimate stress and strain via absorbing energy [39]. Combined with technology advantages of 3D printing, the bionic feather design can be used in design and preparation of continuous carbon fiber-reinforced polylactic acid composite to solve the enhancement problem of mechanical strength.

In this paper, a series of continuous carbon fiber-reinforced PLA composites were prepared by 3D printing. By modifying the printing path program, the 3D printing composites with vertical carbon fiber structure and bionic feather structure were successfully prepared. The tensile strength and impact toughness of 3D printed composites with different arrangements of carbon fibers were investigated to exhibit effects of bionic design on mechanical strength. The mechanical mechanisms of bionic design were also disclosed to guide the application of bionic composites.

## 2. Experiment

### 2.1. Materials

The feathers were observed after obtaining them from an eagle-owl specimen. 1 K continuous carbon fiber which comprised of 1000 single carbon fiber filaments was purchased from Toray Industries, Inc., Tokyo, Japan. The model of those carbon fiber is T300, and it belongs to the HT carbon fiber. Polylactic acid (PLA) printing consumables with a density of 1.24 g/mL was purchased from Sanweicube Co., Ltd., Shen Zhen, China.

### 2.2. 3D Printing of Bionic Composite

**Figure 1:**
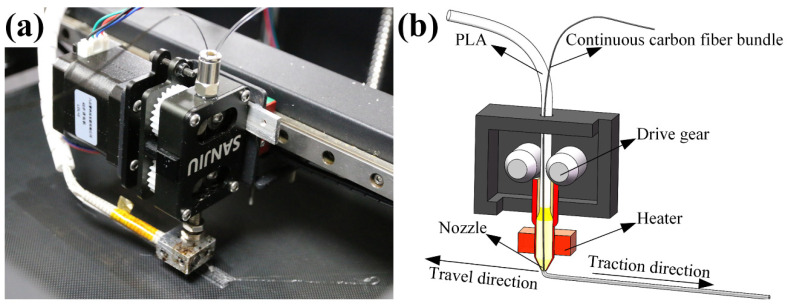
(**a**) Entity diagram and (**b**) structure diagram of 3D printer extrusion head.

Figure 1a shows the proximal extrusion head structure of a 3D printing machine was purchased from Industrial Six point zero Technology Development Co., Ltd. Guangzhou, China. As shown in Figure 1b, the extrusion head of the 3D printer was composed of an extruder, a heating head and a nozzle. Through the driving gear in the extruder, the PLA and continuous carbon fiber were squeezed into the heating head with a temperature of 212 °C ± 5 °C. During the printing process, the continuous carbon fiber was always being wrapped by the PLA with molten state. With ceaselessly being squeezed into the heating head, the continuous carbon fiber wrapped with PLA was extruded from the nozzle to the printing platform. In order to prevent the printing nozzle becoming clogged by carbon fiber during the printing process, a cone-shaped nozzle with a diameter of 0.8 mm was selected.

Before 3D printing, STL files were generated by commercial 3D software SolidWorks (Dassault Systemes S.A., Waltham, MA, USA). The original G code files were obtained through slicing those STL files by the open source 3D slice software Slic3r. Due to special bionic 3D printing structure, those G code files cannot be generated by commercial slice software. The commercial 3D printing software Repetier-Host (Hot-World GmbH & Co. KG, Knickelsdorf, Germany) was used to preview and modify those G code files.

In order to verify the effect of the bionic feather structure on the strength of the 3D printing samples, three kinds of structures were designed and named as Model I, Model II and Model III, respectively. The number of printed layers of all samples were ten. The height of each layer was 0.2 mm ± 0.01 mm. The printing temperature of the first layer of three models was 215 °C ± 5 °C. The subsequent printing temperature of other layers was 212 °C ± 5 °C. The heating platform temperature was 60 °C ± 3 °C. The first printing layer cooling fan power was 0%. The second printing layer cooling fan power was 50%. The subsequent cooling fan power of other layers was 100%. The printing fill rate was 65% ± 3%. The printing speed of the first layer was 10 mm/min. Printing speed of other layers was 15 mm/min.

### 2.3. Characteristics

#### 2.3.1. Microstructure

Before the observation of the microstructure of Model II samples and Model III samples to analyze the reinforcement mechanism of fractured carbon fiber on PLA via a scanning electron microscope (SEM) (EVO 18 Carl Zeiss, Oberkochen, Germany), the section of the corresponding sample was produced by a tensile test or impact test.

#### 2.3.2. Tensile Strength

In order to investigate the effects of continuous carbon fibers and different 3D structures on the stress of PLA resin samples, the size of samples was 75 mm × 10 mm × 2 mm (length × width × thickness). The tolerances of length and width are all within ±0.4 mm, and the tolerances of height within ±0.1 mm. For each model (Model I, Model II and Model III), 5 identical samples were used for the same test. A total of 15 samples were used for the tensile test. The universal testing machine (Model C43, MTS Criterion, Eden Prairie, MN, USA) was chosen for testing. The constant loading rate was 1 mm/min, and 5 samples were tested to calculate the average value of tensile strength.

#### 2.3.3. Impact Toughness

In order to study the effects of continuous carbon fibers and different printing structures on the toughness of PLA resin samples, the size of samples was 75 mm × 10 mm × 2 mm (length × width × thickness). The tolerances of length and width are all within ±0.4 mm, and the tolerances of height within ± 0.1 mm. For each model (Model I, Model II and Model III), 5 identical samples were used for the same test. A total of 15 samples were used for the impact test. The simply supported beam impact testing machine (JC-50D, Beijing Times Peak Technology Co., Ltd., Beijing, China) was selected for testing. The 7.5 J pendulum was used for testing, and 5 samples were tested to calculate the average value of impact toughness.

## 3. Results and Discussion

### 3.1. Bionic Structure Design

**Figure 2:**
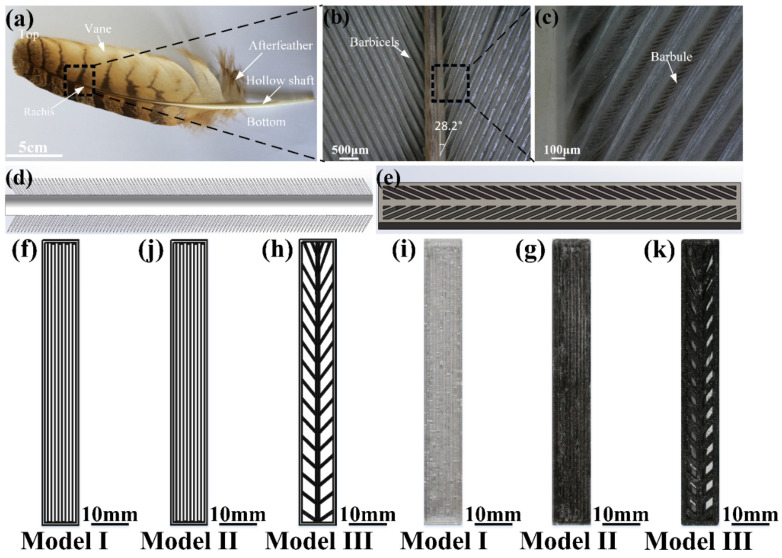
(**a**) Morphology, (**b**) barbicels, (**c**) barbules of eagle-owl feather, (**d**) bionic simplified model and (**e**) bionic structure model of feather, 3D printing path of (**f**) Model I, (**j**) Model II and (**h**) Model III and 3D printing sample of (**i**) Model I, (**g**) Model II and (**k**) Model III.

As shown in Figure 2a–c, the eagle-owl feather was constituted by a hollow shaft, afterfeather, rachis and vane. The vane of bird feathers were composed of many barbicels and barbules. From the top to the bottom of the feather, the angles between the rachis and the barbicels were increased. Compared with the unidirectionally arranged continuous carbon fiber-reinforced composite material, the composite material with a special angle owned higher ultimate stress and strain, which can absorb more energy. [39] As shown in Figure 2d,e, based on the feather, the bionic structure model was built. In order to investigate the effects of addition of continuous carbon fiber and bionic structure model on mechanical strength, three kinds of models include Model I, Model II and Model III were designed, as shown in Figure 2f–k. Figure 2f shows the printing structure of vertical configuration (Model I) with sample size of 75 mm × 10 mm × 2 mm (length × width × height). The Model I sample was only constituted with PLA resin and without carbon fiber. The printed sample of Model I was shown in Figure 2i. Figure 2j shows the printing structure of vertical configuration (Model II) with sample size of 75 mm × 10 mm × 2 mm (length × width × height). Samples of Model II were printed with PLA resin and continuous carbon fiber. The printed Model II was shown in Figure 2g. Model I and Model II were composed of 8 3D printing line segments. The two ends of the line segment were connected to the frame. Figure 2h shows the bionic feather structure configuration (Model III) with sample size of 75 mm × 10 mm × 2 mm (length × width × height). The printed sample of Model III was shown in Figure 2k. According to the designing 3D printing parameters and the path of the bionic feather structure, the 3D printing of the bionic feather structure composite was realized. The bionic feather structure was composed of rachis structure arranged vertically in the middle and barbicels structure. The rachis structure was composed of three vertical 3D printing line segments. The barbicels structure was composed of two 3D printing line segments. From the top to the bottom of the 3D printed samples, the angle between rachis and barbicels increased gradually. The long side of the sample was 75 mm ± 0.4 mm, the wide side of the sample was 10 mm ± 0.4 mm, and the thickness of the sample was 2 mm ± 0.1 mm. The mass of printed samples were in the range of 1.56 g ± 0.08 g. The mass fraction of continuous carbon fiber was 10.5 wt% ± 0.6 wt%. The structure data of models are listed in Table 1 in detail.

### 3.2. Mechanical Strength

**Figure 3:**
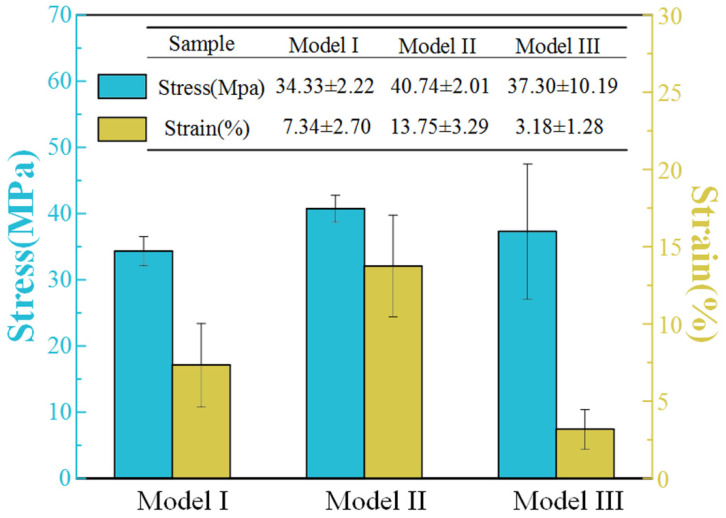
The tensile strength of Model I, Model II, Model III.

The tensile strength histograms of Model I, Model II and Model III are shown in Figure 3. The stress values of the tensile strength of Model I, Model II and Model III were 34.33 Mpa ± 2.22 Mpa, 40.74 MPa ± 2.01 Mpa, and 37.30 MPa ± 10.19 Mpa, respectively. The strain values of Model I, Model II and Model III were 7.34% ± 2.70%, 13.75% ± 3.29%, and 3.18% ± 1.28%, respectively. Comparing the average stress value, under the same 3D structure, the tensile strength of Model II was 18.7% stronger than the Model I. By adding continuous carbon fiber, the tensile strength of the 3D printing sample was improved. The content of continuous carbon fibers which were paralleled to tensile force in Model III was lower than that of Model II, which resulted in the relatively low tensile strength of Model III. However, the stress of Model III was higher than that of Model I. Compared with the tensile stress and strain of PLA matrix, the bionic continuous carbon fiber-reinforced PLA composite realized the purpose of bionic design, which proved the feasibility of bionic design and 3D printing method. Under the conditions of consistency of 3D printing parameters including weight of sample and continuous carbon fiber, the tensile strength was restricted. Combined with the adjustability of 3D printing technology, the tensile strength of bionic composite can be improved further.

The impact toughness histograms of Model I, Model II and Model III are shown in Figure 3. The impact toughness of Model I, Model II and Model III were 339.115 KJ/m^2^ ± 18.203 J/m^2^, 435.389 KJ/m^2^ ± 7.144 J/m^2^, and 691.597 KJ/m^2^ ± 3.565 J/m^2^, respectively. The impact toughness of Model II was 28% higher than that of Model I. The addition of continuous carbon fiber enhanced impact toughness of 3D printing composite under the same structure. The impact toughness of Model III was 103.9% higher than that of Model I. The impact toughness of the bionic composite was improved. The sample with the bionic feather structure (Model III) owned barbicels structure with various angles. During the impact process, after the impact force was acted at the outer frame of the sample, the impact load was transferred to the barbicels structure and the rachis structure. Then, the load was transferred to the other half of the bionic feather structure. Due to this, the angles were decreased between barbicels structure and load, and the impact energy was consumed.

**Figure 4:**
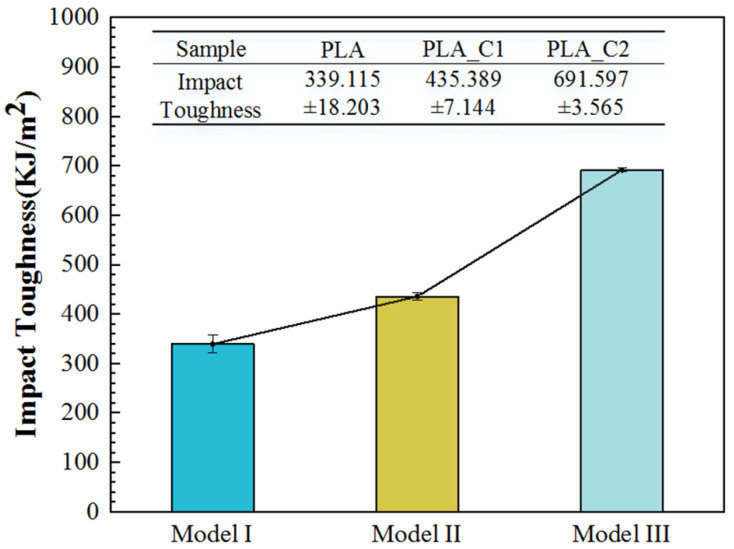
The impact toughness of Model I, Model II, Model III.

Combined with Figure 3 and Figure 4, it can be found that the mechanical properties of the 3D printing bionic composite were enhanced. Even though the tensile strength of bionic composite was restricted, the impact toughness was effectively enhanced. Therefore, the bionic composites can be emphatically applied to areas with large impact loads.

### 3.3. Microstructure Analysis

**Figure 5:**
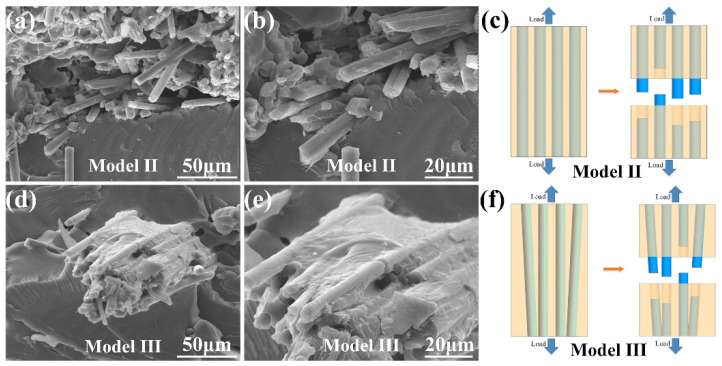
The microstructure of the tensile test sample of (**a**,**b**) Model II, (**c**) the schematic diagram of tensile fracture of Model II. The microstructure of the tensile test sample of (**d**,**e**) Model III, (**f**) the schematic diagram of tensile fracture of Model III.

The microstructure of the tensile fracture surfaces of Model II and Model III were exhibited in Figure 5. The continuous carbon fibers in different 3D printing structures were wrapped by PLA resin. Figure 5a,b were the vertical structure sample (Model II). The fracture section of the matrix part was relatively flat. The carbon fiber was pulled out or fractured, which consumed external stress on the base of effective bond strength between the carbon fiber and PLA resin matrix. Figure 5c,d were the bionic 3D printing composite (Model III). A bunch of continuous carbon fiber were not perpendicular to the fracture surface. Compared with Model II, the continuous carbon fiber in the bionic composite was not parallel to the force direction. Due to the angle, the continuous carbon fiber and load were decreased, and a part of the load were consumed. As a result, the stiffness and strength in the off-axis direction were reduced [40]. Therefore, the tensile performance of the bionic composite was slightly lower than that of the vertical structure.

**Figure 6:**
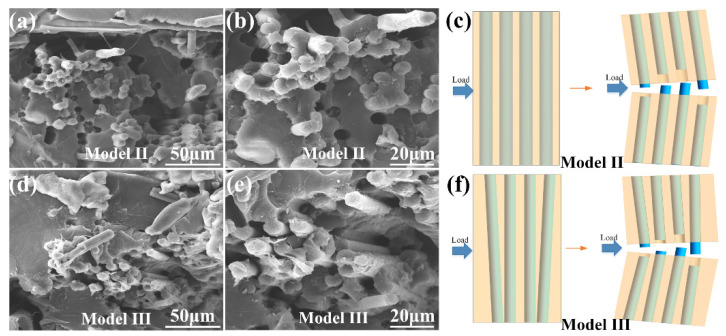
The microstructure of the impact test sample of (**a**,**b**) Model II, (**c**) The schematic diagram of impact fracture of Model II. The microstructure of the impact test sample of (**d**,**e**) Model III, (**f**) The schematic diagram of impact fracture of Model III.

As shown in Figure 6, the microstructure of the impact fracture surfaces of Model II and Model III were exhibited. Due to fracture and pulling out of the continuous carbon fiber, the load was effectively transferred from the PLA resin matrix to the carbon fibers, which consumed impact force. The direction of load was perpendicular to the carbon fiber, the crack propagation was the primary which caused sample fracture. Crack failure in the matrix was hindered by continuous carbon fibers with different alignment angles [41]. Compared with continuous carbon fibers arranged in a vertical direction, the bionic composite with feather structure performed higher crack arrest property.

Combined with Figure 5 and Figure 6, the addition of continuous carbon fiber improved the mechanical strength of PLA matrix, which proved the feasibility of 3D printing. Under conditions of tensile and impact forces, those angles between the feather barbicel structure and the rachis structure consumed the external forces. When the sample was damaged by external force, Model III with different fiber arrangement angles prevented the extension of cracks. Therefore, through the bionic structure design and composite material 3D printing, the performance of 3D printed samples has been enhanced, which expanded the application field of 3D printing samples.

## 4. Conclusions

In this paper, inspired by the arrangement structure of the feathers of eagle-owl feather with characteristics of light weight and high strength, the bionic continuous carbon fiber-reinforced PLA composite were designed and 3D printed. From the top to the bottom of the feather, the angles between the rachis and the barbicels were increased. The designed bionic structure model exhibited angle arrangement of feather. 3D printing realized the reappearing of bionic structure design in bionic composites. Bionic design with specific structure arrangement enhanced mechanical properties including tensile strength and impact toughness of bionic composite. Continuous carbon fiber bonded tightly with PLA matrix. Due to the broken fiber on cross-sections of bionic composite, the load was effectively transferred from the PLA matrix to the carbon fibers. When the external force acted on bionic composite, the propagation of cracks was hindered on the base of different angle arrangement of continuous carbon fibers in PLA matrix. The content of continuous carbon fibers which were paralleled to tensile force in bionic composite was lower than that of composite with vertical arrangement structure of continuous carbon fiber, which resulted in the relatively low tensile strength of bionic composite. However, the bionic composite exhibited high impact resistance property on the base of bionic feather structure, which proved the feasibility and effectiveness of bionic design and 3D printing. The fracture and pull out of continuous carbon fiber were the main mechanical mechanisms of bionic composite, which provided a new design and preparation method for the application of carbon-reinforced resin composite.

## Figures and Tables

**Table 1:** Three types of print model parameters.

	Model I	Model II	Model III
Printing Materials	PLA	PLA and continuous carbon fiber	PLA and continuous carbon fiber
3D Structure	vertical	vertical	bionic feather structure
Length	75 mm ± 0.4 mm	75 mm ± 0.4 mm	75 mm ± 0.4 mm
Width	10 mm ± 0.4 mm	10 mm ± 0.4 mm	10 mm ± 0.4 mm
Height	2 mm ± 0.1 mm	2 mm ± 0.1 mm	2 mm ± 0.1 mm

## Data Availability

The data presented in this study are available on request from the corresponding author.
